# Rare X-Linked Hypohidrotic Ectodermal Dysplasia in Females Associated with *Ectodysplasin-A* Variants and the X-Chromosome Inactivation Pattern

**DOI:** 10.3390/diagnostics12102300

**Published:** 2022-09-23

**Authors:** Haochen Liu, Lanxin Su, Hangbo Liu, Jinglei Zheng, Hailan Feng, Yang Liu, Miao Yu, Dong Han

**Affiliations:** Department of Prosthodontics, Peking University School and Hospital of Stomatology & National Center of Stomatology & National Clinical Research Center for Oral Diseases & National Engineering Research Center of Oral Biomaterials and Digital Medical Devices, Beijing 100081, China

**Keywords:** *Ectodysplasin-A* (*EDA*), skewed X-chromosome inactivation, X-linked hypohidrotic ectodermal dysplasia (XLHED), phenotypic analysis

## Abstract

The goal of this study was to identify the pathogenic gene variants in female patients with severe X-linked hypohidrotic ectodermal dysplasia (XLHED). Whole-exome sequencing (WES) and Sanger sequencing were used to screen for the pathogenic gene variants. The harmfulness of these variations was predicted by bioinformatics. Then, skewed X-chromosome inactivation (XCI) was measured by PCR analysis of the CAG repeat region in the human *androgen receptor* (*AR*) gene in peripheral blood cells. Two novel *Ectodysplasin-A* (*EDA*) heterozygous variants (c.588_606del19bp and c.837G>A) and one heterozygous variant (c.1045G>A, rs132630317) were identified in the three female XLHED patients. The bioinformatics analysis showed that these variants might be pathogenic. The tertiary structure analysis showed that these variants could cause structural damage to EDA proteins. Analysis of the skewed X-chromosome inactivation revealed that extreme skewed X-chromosome inactivation was found in patient #35 (98:2), whereas it was comparatively moderate in patients #347 and #204 (21:79 and 30:70). Our results broaden the variation spectrum of *EDA* and the phenotype spectrum of XLHED, which could help with clinical diagnosis, treatment, and genetic counseling.

## 1. Introduction

Ectodermal dysplasia (ED) is a large and heterogeneous group of congenital disorders characterized by alterations in two or more ectodermal structures, including at least one in the hair, teeth, nails, or sweat glands [1]. Hypohidrotic ectodermal dysplasia (HED) is the most common type of ED. HED is characterized by a triad of signs comprising sparse hair (hypotrichosis), abnormal or missing teeth (anodontia or hypodontia), and the inability to sweat (anhidrosis or hypohidrosis). At present, there are three clear genetic inheritance modes of HED: autosomal dominant, autosomal recessive, and X-linked inheritance [2]. Variants in the Eda receptor (*EDAR*, OMIM*604095), which is located on chromosome 2q11-q13, or in the Edar-associated death domain (*EDARADD*, OMIM*606603), which is located on chromosome 1q42-q43, have been implicated in the autosomal recessive and dominant HED forms, respectively [3]. Variants in ectodysplasin-A (*EDA*, OMIM*300451), which is located on chromosome Xq12-q13.3, have been identified in X-linked HED (XLHED, OMIM#305100) [4].

XLHED is considered to be the most frequently occurring form of HED [2], and males are more susceptible due to the hemizygous state of their X chromosome [5,6,7]. Most females with pathogenic heterozygous variants in *EDA*, which are due to the presence of a second normal allele on the X chromosome, only present with mild phenotypes or “partial” involvement, often meaning they are not referred to physicians [8]. So far, a few studies have reported on how heterozygous *EDA* pathogenic variants can also cause severe XLHED in females [9,10,11]; however, the corresponding clinical evidence needs to be expanded, and the pathogenic mechanisms require further study.

In this study, we report on three rare female patients with severe XLHED carrying heterozygous *EDA* pathogenic variants and discuss the situation of skewed X-chromosome inactivation (XCI) in these patients. The results of our study might help to broaden the variant spectrum of *EDA* and the phenotype spectrum of XLHED.

## 2. Materials and Methods

### 2.1. Subjects

Three unrelated females with clinical signs of ectodermal derivative impairment and their available family members were recruited from the Department of Prosthodontics at the Peking University School and Hospital of Stomatology. The patients and available family members were examined for possible clinical symptoms in the hair, skin, nails, and intra-oral region. Diagnoses of hypohidrosis or anhidrosis were based on the dermatology records, clinical observations, and descriptions of the patients and their families. Their tooth agenesis patterns were diagnosed by an experienced dentist. Written informed consent was obtained from all participants for the use of their blood and clinical data and for the publication of their photographs. This study was approved by the Ethics Committee of the Peking University School and Hospital of Stomatology: PKUSSIRB-202162021(22 February 2021).

### 2.2. Variants Analysis

Peripheral blood samples were obtained from patients and their available family members. Total genomic DNA was extracted using the protocol recommended by the BioTek DNA Whole-Blood Mini Kit (BioTek, Beijing, China). Whole-exome sequencing (WES) was performed by Beijing Angen Gene Medicine Technology (Beijing, China) using the Illumina-X10 platform to screen for the potential pathogenic variants. The identification processes for the pathogenic variants were as follows: (a) Orodental-related development-related genes were analyzed [12]. (b) We excluded variants with minor allele frequency (MAF) ≥ 0.01 in more than one of four frequency databases: the single nucleotide polymorphism database (dbSNP, http://www.ncbi.nlm.nih.gov/projects/SNP/snp_summary.cgi (accessed on 23 July 2022)), the Genome Aggregation Database (gnomAD, http://gnomad.broadinstitute.org (accessed on 23 July 2022)), the Exome Aggregation Consortium (ExAC, http://exac.broadinstitute.org (accessed on 23 July 2022)), and the 1000 Genomes Project database in Ensembl (http://asia.ensembl.org/Homosapiens/Info/Inde (accessed on 23 July 2022)). (c) Bioinformatics analysis was implemented for the remaining variants to predict the functional impact using Mutation Taster (http://www.mutationtaster.org (accessed on 23 July 2022)), Functional Analysis through Hidden Markov Models v2.3 (Fathmm, http://fathmm.biocompute.org.uk (accessed on 23 July 2022)), and Polymorphism Phenotyping v2 (PolyPhen-2, http://genetics.bwh.harvard.edu/pph2/ (accessed on 23 July 2022)).

To verify the WES results, *EDA* (NM_001399.5) genes were sequenced using Sanger sequencing. The related exons of the *EDA* gene and the intron–exon boundaries were amplified by polymerase chain reaction (PCR) with specific primers (Supplementary Table S1). The PCR products were sequenced by Tsingke Biological Technology (Beijing, China).

### 2.3. Conservation and Protein Conformational Analyses

For conservation analysis, the amino acid sequences of EDA (NP_001390.1) for eight different species were obtained from the UniProt (https://www.uniprot.org/ (accessed on 17 September 2022)). Molecular Evolutionary Genetics Analysis Version 11 (MEGA 11.0.11, Tamura, Stecher, and Kumar, Philadelphia, PA, USA) was used to conduct multiple sequence alignment [13].

The protein tertiary structure of EDA (PDB ID 1RJ7; X-ray, resolution 2.3Å) was obtained from the RCSB Protein Data Bank (RCSB PDB, https://www.rcsb.org/ (accessed on 17 September 2022)). PyMol v2.1(Molecular Graphics System, DeLano Scientific, San Carlos, CA, USA) was used to analyze the changes in the EDA tertiary structure.

### 2.4. Analysis of XCI

The human *androgen receptor* (*AR*) gene consists of a varying number of CAG repeats in exon 1 and has been used as a marker of skewed XCI through differential PCR amplification following digestion with the methylation-sensitive restriction enzyme HpaII [14]. In brief, PCR (forward primer sequence: TCCAGAATCTGTTCCAGAGCGTGC; reverse primer sequence: GCTGTGAAGGTTGCTGTTCCTCAT) including the polymorphic CAG_n_ repeat was performed before and after digestion with the methyl-sensitive restriction enzyme HpaII. Fragment analysis was employed to determine the size and qualitative distribution of the PCR products. The products were further analyzed by capillary electrophoresis. XCI was considered to be skewed (non-random) if the ratio of the two alleles exceeded 70:30.

## 3. Results

### 3.1. Clinical Findings

**Figure 1:**
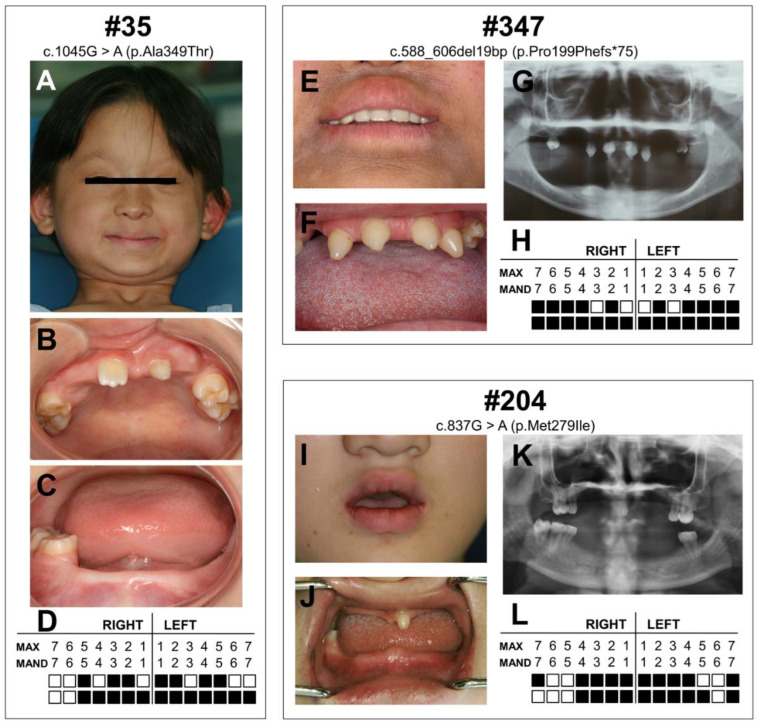
Clinical features of patients with X-linked hypohidrotic ectodermal dysplasia (XLHED). (**A**) Facial photographs of patient #35. (**B**,**C**) Intraoral photographs of patient #35. (**D**) Schematic of congenitally missing permanent teeth of patient #35. According to medical records, right maxillary first premolar, right maxillary second molar, left maxillary canine, and right mandibular second molar were in the jawbone and could be found by panoramic radiograph. (**E**) Perioral photographs of patient #347. The patient wore removable dentures. (**F**) Intraoral photographs of patient #347. (**G**,**H**) Panoramic radiograph and schematic of congenitally missing permanent teeth of patient #347. (**I**) Perioral photographs of patient #204. (**J**) Intraoral photographs of patient #204. (**K**,**L**) Panoramic radiograph and schematic of congenitally missing permanent teeth of patient #204. Solid squares indicate congenitally missing teeth. Max: indicates maxillary. Mand: mandibular.

The three patients exhibited variability in the phenotypic severity of XLHED. The clinical and radiographic information is presented in Figure 1 and is summarized in Table 1.

Patient #35 was an 8-year-old girl who presented with 19 congenitally missing permanent teeth, sparse eyebrows, hyperpigmentation, maxillary hypoplasia, ‘saddle’ nose, prominent lips, perioral and periocular wrinkles, dry skin, and hypohidrosis (Figure 1A–D). Her parents did not have any obvious phenotypic abnormalities. Her parents all denied the existence of a family history of HED or tooth agenesis.

Patient #347 was a 21-year-old female. She had 24 congenitally missing permanent teeth, sparse scaly hair, sparse eyebrows, erythematous patches, hyperpigmentation, ‘saddle’ nose, prominent lips, perioral and periocular wrinkles, anhidrosis, xerostomia, and dry eyes (Figure 1E–H). Her father did not have any obvious phenotypic abnormalities. According to her father’s description, her mother was normal, and there was no family history.

Patient #204 was a 14-year-old girl presenting with congenital agenesis of 20 permanent teeth. She had sparse scaly and thin hair, sparse eyebrows, erythematous patches, hypohidrosis, dry skin, and dry eyes (Figure 1I–L). No clinical symptoms were observed in her parents, and we could not find any family history of HED or tooth agenesis.

### 3.2. EDA Variants

**Figure 2:**
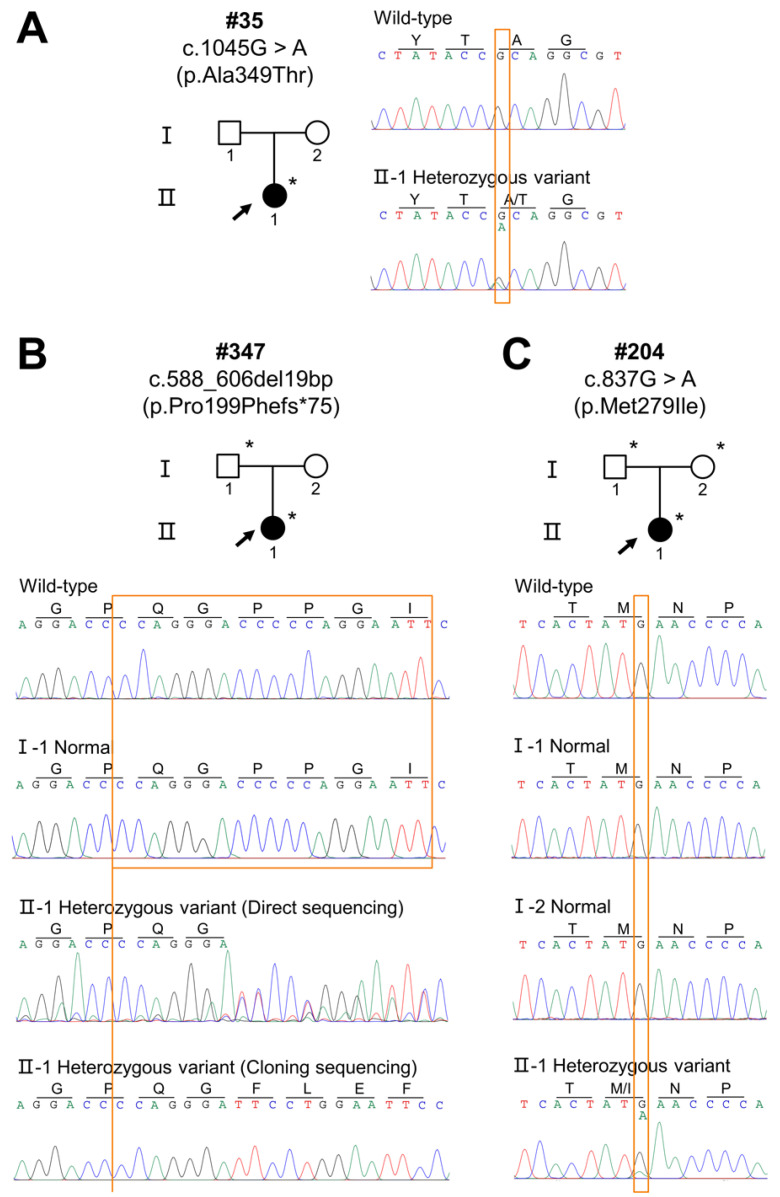
Sequencing chromatograms of three female patients with XLHED. (**A**) Sequencing chromatograms show a heterozygous missense variant (c.1045G>A, p.Ala349Thr) identified in patient #35. (**B**) Sequencing chromatograms show a heterozygous frameshift variant (c.588_606del19bp, p.Pro199Phefs*75) identified in patient #347. (**C**) Sequencing chromatograms show a heterozygous missense variant (c.837G>A, p.Met279Ile) identified in patient #204. Black arrows indicate the proband in each family. Solid circles and squares represent the individuals with HED. Asterisks indicates that DNA samples are available. Black circles represent the individuals with XLHED. Black arrows indicate the probands.

Two novel *EDA* heterozygous variants (c.588_606del19bp and c.837G>A) and one reported heterozygous variant (c.1045G>A, rs132630317) were identified. Patient #35 carried the heterozygous missense variant, c.1045G>A (p.Ala349Thr) (Figure 2A). Unfortunately, we did not obtain DNA samples from her parents and could not determine the source of this variation. Patient #347 carried the novel heterozygous frameshift variant c.588_606del19bp (p.Pro199Phefs*75), and her father ‘s genotype was normal (Figure 2B). The DNA sample of her mother was unavailable, so we were unable to identify whether this variant was de novo. Patient #204 carried the novel heterozygous missense variant c.837G>A (p.Met279Ile), and her parents’ genotypes were normal (Figure 2C), thus confirming that patient #204’s *EDA* variant was de novo.

### 3.3. Bioinformatics Findings

**Figure 3:**
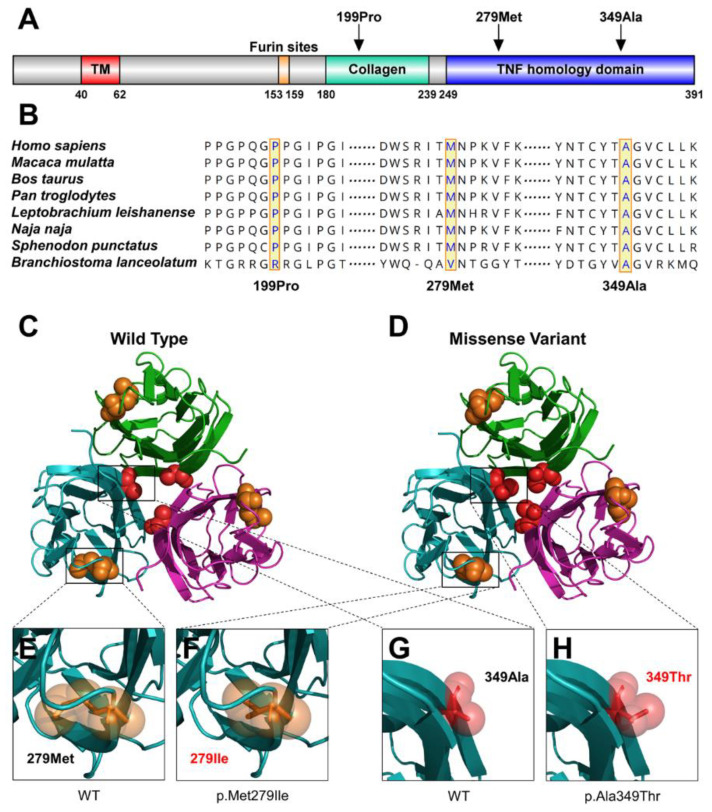
Bioinformatics analysis of the Ectodysplasin-A (EDA) variants. (**A**) Schematic diagram of the wild-type EDA protein and the localization of the three EDA variants identified in this study. (**B**) Conservation analysis of the EDA amino acid sequences among different species. (**C**) Structure of wild-type EDA. (**D**) Structure of p.Met279Ile and p.Ala349Thr in EDA. (**E**) Magnified structure of 279Met in EDA. (**F**) Magnified structure of p.Met279Ile in EDA. (**G**) Magnified structure of 349Ala in EDA. (**H**) Magnified structure of p.Ala349Thr in EDA.

We conducted bioinformatics analyses to predict the functional effects of the three identified *EDA* variants. Based on the results of conservation analyses among multiple species, 199Pro was located in the highly conserved collagen domain, and 279Met and 349Ala were located in the highly conserved TNF homology domain (Figure 3A,B).

These three variants caused varying degrees of conformational changes in the EDA protein (Figure 3C,D). For p.Met279Ile, the residue at sequence position 279 is a hydrophobic methionine with an aliphatic side chain. The variant resulted in the methionine being substituted with a hydrophobic isoleucine with an aliphatic side chain (Figure 3E,F). For p.Ala349Thr, the residue at sequence position 349 is a hydrophobic alanine with an aliphatic side chain; the variant residue was threonine with a neutral side chain (Figure 3G,H). For p.Pro199Phefs*75 starting in the collagen domain, the variant led to the premature termination at residue 199Pro and resulted in a lack of the EDAR-binding domain, which is essential for stimulating EDAR.

Moreover, according to the classification of pathogenic variants with the standards outlined by the 2015 American College of Medical Genetics and Genomics and the Association for Molecular Pathology [15], all three variants were predicted to be pathogenic (Table 2).

### 3.4. Skewed XCI

**Figure 4:**
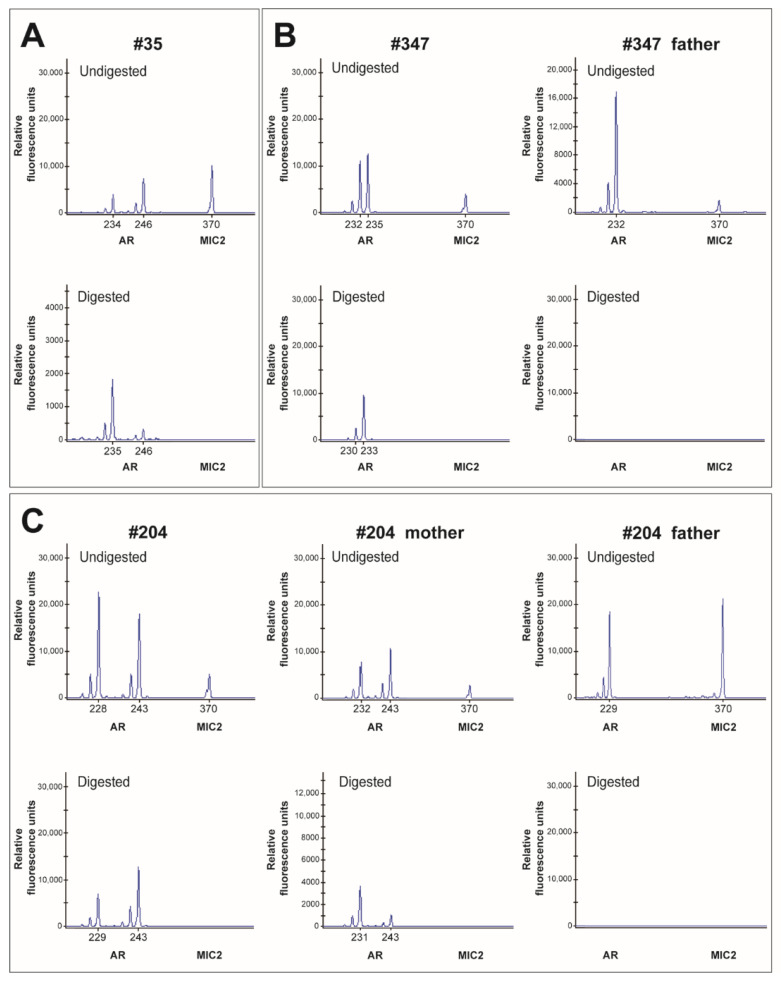
Skew of the X-chromosome inactivation (XCI) analysis. (**A**) The capillary electrophoresis results of patient #35. (**B**) The capillary electrophoresis results of patient #347 and her father. (**C**) The capillary electrophoresis results of patient #204 and her parents.

Results from the XCI analysis, as measured by androgen receptor allele methylation, revealed that patient #35 had extremely skewed (98:2) XCI (Figure 4A). Unfortunately, the origin of the chromosomes could not be determined because there were no DNA samples from her parents. Patient #347 showed moderately skewed (21:79) XCI, and the maternal source X chromosome presented a higher inactivation rate (Figure 4B). For patient #204, the XCI results also revealed moderate skew (30:70) with a higher inactivation rate of the maternally sourced X chromosome (Figure 4C). The *MIC2* gene (370 bp) was used as an internal control.

## 4. Discussion

In this study, we assessed three female patients suffering from severe XLHED. Through WES and Sanger sequencing, we identified heterozygous pathogenic variants in the *EDA* gene among these patients: two novel *EDA* heterozygous variants (c.588_606del19bp and c.837G>A) and one heterozygous *EDA* variant (c.1045G>A, rs132630317). Based on genetic co-segregation, the missense variant c.837G>A carried by patient #204 was a de novo variant. However, since the DNA samples from the parents of patient #35 and the mother of patient #347 were not obtained, we were unable to determine the source of inheritance or a genetic model of the two families.

The variant *EDA*: c.1045G>A (p.Ala349Thr) has been widely found in HED patients from multiple ethnic populations [4,16,17,18,19,20,21] at what is thought to be a ‘hot spot’ on the *EDA* gene. In 2004, Na et al. reported a 6-year-old boy with XLHED carrying this variant [19]. The boy presented with sparse hair, the absence of sweat and sebaceous glands, oligodontia, frontal bossing, and a prominent supraorbital ridge with periorbital hyperpigmentation [19]. In 2019, Park et al. reported a 9-year-old boy with XLHED carrying this variant [17]. The boy also presented with the typical XLHED phenotypes: sparse hair, oligodontia, anhidrosis, dry skin, dermatitis/eczema, and periorbital wrinkles [17]. However, all previous reports were of male patients. In this study, patient #35 had the *EDA*: c.1045G>A (p.Ala349Thr) variant and showed a similar clinical phenotypes: 19 congenitally missing permanent teeth, sparse eyebrows, dry skin and hypohidrosis, hyperpigmentation, perioral and periocular wrinkles, maxillary hypoplasia, ‘saddle’ nose, and prominent lips. Therefore, this study was the first to report severe XLHED in female carriers of the variant c.1045G>A in *EDA*.

The *EDA* frameshift variant c.588_606del19bp (p.Pro199Phefs*75) is a previously unreported variant. This variant leads to a premature termination at residue 199, which, in turn, results in the deletion of the important TNF homology domain of EDA. The TNF homology domain, which mediates binding to EDAR, was totally missing in this variant. As a result, the EDA−EDAR axis would be disrupted and would lead to XLHED (in males). Conservative analysis and bioinformatics prediction showed that this variant was pathogenic. Our previous study showed that more than half of the variants in the XLHED subjects were speculated to be loss of function variants, such as nonsense, insertion, and deletion variants [6]. Variants that severely affect the function of EDA proteins are more likely to cause severe XLHED symptoms.

The *EDA* c.837G>A (p.Met279Ile) variant, which was identified in patient #204 and who had severe XLHED, is also a novel pathogenic variant, as confirmed by conservative analysis and bioinformatics prediction. This variant is located in the highly conserved TNF homology domain, which might impair the specific binding of EDA to EDAR [22,23]. Therefore, we assume that these two novel variants (c.588_606del19bp and c.837G>A) may result in pathogenicity.

XCI is a process associated with the pathogenesis of several diseases [24]. It involves the random transcriptional silencing of one of the two X chromosomes in female somatic cells and occurs in the early blastocyst stage [25]. Once X-inactivation is initiated, the selected X chromosome remains inactive during the remaining life of the cell, and the X-inactivation mode is transmitted to its daughter cells. According to logic, XCI randomness results in a coexistence of both chromosomes activated in a 1:1 ratio. This is particularly important in terms of phenotypic changes, because in 50% of female cells, variants that could cause major difficulties in men are compensated for by another chromosome [26]. This random process follows a normal distribution, which almost inevitably leads to a very unbalanced ratio [24]. The existence of skewed XCI, especially in extreme cases (over 90% of the cells with inactivation of the same chromosome), allows the development of multiple diseases in noncommon forms that are mainly X-linked diseases in women.

Several studies have shown that skewed XCI can lead to severe HED [10,11,27]. In 2007, Karen et al. reported a girl with severe XLHED and normal mental development and who had completely skewed XCI and only the paternal X active in her peripheral blood cells [10]. In 2012, Pavlovsky et al. found that only the variant allele was present in a hairless patch in a female proband with severe XLHED [11]. In 2018, Lei et al. reported a novel 1-bp deletion variation in *EDA* and extremely skewed XCI causing severe XLHED in a Chinese girl [27]. Controversially, some studies have not supported this view. It has been reported that the HED phenotype in *EDA* female carriers is not correlated with the XCI pattern [28]. Laura et al. found that women with mild HED did not have higher non-randomized XCI ratios and that there was no significant correlation between tilted XI ratios and XLHED phenotypic expression levels [29]. In this study, patient #35 showed extremely skewed (98:2) XCI, and patients #347 and #204 showed moderately skewed (21:79 and 30:70) XCI. We did not find a distinct association between the disease phenotype and the XCI pattern. There are two possible reasons for this result: (I) Tissue-specific variations of XCI ratios cannot be excluded. In some cases, there is extensive variation in XCI ratios between various tissue types. [30]. In this study, we used DNA from peripheral blood leukocytes. DNA from skin tissue or from teeth may have provided different results. (II) XCI in the human placenta is patchy and distinct from XCI in adult tissues [31]. We cannot exclude differences in the results due to time specificity. Therefore, the precise mechanisms of XCI in female carriers of *EDA* variants are not clear, and more studies are needed to elaborate the etiology of female patients with XLHED caused by heterozygous *EDA* pathogenic variants.

Taken together, we reported on three rare female patients with severe XLHED caused by two novel and one *EDA* heterozygous variant. Our results broaden the variation spectrum of *EDA* and the phenotype spectrum of XLHED, which could help with clinical diagnosis, treatment, and genetic counseling. However, the pathogenic mechanism of *EDA* heterozygous variants associated XLHED in female patients is not clear and needs to be further studied.

## Figures and Tables

**Table 1:** Overview of the clinical features of the patients in this study.

Clinical Features	#35	#347	#204
Gender	Female	Female	Female
Age (years)	8	21	14
Permanent dentition			
Missing number (excluding third molar, *n* = 28)	19	24	20
Peg-shaped teeth	−	+	−
Hair			
Sparse scalp hair	−	+, brown	+, thin
Sparse eyebrows	+	+	+
Skin			
Erythematous patches	−	+	+
Hyperpigmentation	+	+	−
Perioral and periocular wrinkles	+	+	−
Dry skin	+	+	+
Gland function			
Hypohidrosis	+	−	+
Anhidrosis	−	+	−
Dry eyes	−	+	+
Xerostomia	−	+	−

+, present; −, absent.

**Table 2:** Pathogenic prediction of the *EDA* variants.

Variant	Patient	Domain	Mutation Taster	Fathmm	PolyPhen-2	gnomAD, dbSNP, 1000G	ACMG Classification (Evidence of Pathogenicity)
c.1045G>A (p.Ala349Thr)	#35	TNF	Disease causing	DAMAGING (−5.92)	Probably damaging (0.996)	rs132630317	Pathogenic PS1 + PM1 + PM2 + PP2 + PP3 + PP4
c.588_606del19bp (p.Pro199Phefs*75)	#347	Collagen	Disease causing	-	-	Not found	Pathogenic PVS1 + PM1 + PM2 + PM4 + PP3 + PP4
c.837G>A (p.Met279Ile)	#204	TNF	Disease causing	DAMAGING (−5.80)	Benign (0.038)	Not found	Pathogenic PS2 + PM1 + PM2 + PP2 + PP3 + PP4

PS1: Same amino acid change as a previously established pathogenic variant regardless of nucleotide change. PM1: Located in a mutational hot spot and/or critical and well-established functional domain (e.g., active site of an enzyme) without benign variation. PM2: Absent from controls (or at extremely low frequency if recessive) in Exome Sequencing Project, 1000 Genomes Project, or Exome Aggregation Consortium. PP2: Missense variant in a gene that has a low rate of benign missense variation and in which missense variants are a common mechanism of disease. PP3: Multiple lines of computational evidence support a deleterious effect on the gene or gene product. PP4: Patient’s phenotype or family history is highly specific for a disease with a single genetic etiology. PVS1: Null variant (nonsense, frameshift, canonical ±1 or 2 splice sites, initiation codon, single or multiexon deletion) in a gene where LOF is a known mechanism of disease. PM4: Protein length changes as a result of in-frame deletions/insertions in a nonrepeat region or stop-loss variants. PP2: De novo (both maternity and paternity confirmed) in a patient with the disease and no family history.

## Data Availability

The variations identified in this study were submitted to the ClinVar database (submission ID: SCV002569098 and SCV002569099).

## Supplementary Materials

The following supporting information can be downloaded at https://www.mdpi.com/article/10.3390/diagnostics12102300/s1: Table S1: Primers Used for PCR Amplification and PCR Conditions.
