# Supplementary material for: Rare X-Linked Hypohidrotic Ectodermal Dysplasia in Females Associated with Ectodysplasin-A Variants and the X-Chromosome Inactivation Pattern
Source: Diagnostics (Basel). 2022 Sep 23;12(10):2300. doi: 10.3390/diagnostics12102300 (PMC9600026; doi:10.3390/diagnostics12102300)
Supplement: Supplementary file 1 [file diagnostics-12-02300-s001.zip › diagnostics-1927832-supplementary.pdf]

Table S1. Primers Used for PCR Amplification and PCR Conditions.

Exon		Primer (5'-3')	Annealing T (°C)
4	Forward	ACAGTACACTCATCACAGGAAAT	60°C
	Reverse	AGTCAGGGAGGGCTTGTAAC	
7	Forward	CCTTCTAGGCTACCCTGGTTG	60°C
	Reverse	CAGGAAGTTAGCCATTGGATG	
8	Forward	AAGAACAATGCCTGTCACCTG	60℃
	Reverse	TTGTCACCCTGGAGTCACTG	
